# Split inactivated COBRA vaccine elicits protective antibodies against H1N1 and H3N2 influenza viruses

**DOI:** 10.1371/journal.pone.0204284

**Published:** 2018-09-28

**Authors:** James D. Allen, Satyajit Ray, Ted M. Ross

**Affiliations:** 1 Center for Vaccines and Immunology, University of Georgia, Athens, GA, United States of America; 2 Department of Infectious Diseases, University of Georgia, Athens, GA, United States of America; 3 Sanofi-Pasteur, Inc., Cambridge, MA, United States of America; Center for Inflammation, Immunity & Infection, Institute for Biomedical Sciences, UNITED STATES

## Abstract

Development of broadly reactive or universal influenza vaccines will be a paradigm shifting event for the influenza vaccine field. These next generation vaccines could replace the current standard of care with vaccines that elicit broadly cross-protective immune responses. However, a variety of *in vitro* and *in vivo* models are necessary to make the best assessments of these vaccine formulations to determine their mechanisms of action, and allow for downselection of candidates prior to human clinical trials. Our group has developed the computationally optimized broadly reactive antigen (COBRA) technology to develop HA head-based strategies to elicit antibodies against H1, H3, and H5 influenza strains. These vaccines elicit broadly reactive antibody responses that neutralize not only historical and contemporary vaccine strains, but also co-circulating variants in mice. In this study, we used H1 and H3 HA antigens in a split, inactivated vaccine (IIV) formulation in combination with the AF03 squalene-in-water emulsion adjuvant in ferrets immunologically naïve to influenza virus. The H3 COBRA IIV vaccine T11 elicited antibodies with HAI activity against more H3N2 influenza strains compared to IIV expressing wild-type H3 HA antigens, except for IIV vaccines expressing the HA from A/Texas/50/2012 (Tx/12) virus. H1 COBRA IIV vaccines, P1 and X6, elicited antibodies that recognized a similar number of H1N1 viruses as those antibodies elicited by IIV expressing the A/California/07/2009 (CA/09) HA. Ferrets vaccinated with the P1 or X6 COBRA IIV were protected against CA/09 challege and cleared virus from the lungs of the ferrets, similar to ferrets vaccinated with the CA/09 IIV.

## Introduction

Influenza vaccination is the most cost-effective method to prevent influenza infections and spread within a community. Current influenza vaccination strategies primarily elicit antibodies that bind to influenza hemagglutinin (HA) and neuraminidase (NA) glycoproteins on the surface of the virus and block viral infection and spread from cell to cell [1, 2]. While live-attenuated and recombinant HA based vaccines are approved for human use, the majority of seasonal influenza vaccines are formulated for the Northern and Southern Hemisphere each year with the majority of vaccine being grown by manufacturers in fertilized chicken eggs. After growth in eggs, influenza virus is harvested from the allantoic fluid and concentrated by zonal ultracentrifugation. Subsequently, the intermediate bulk material is inactivated and formulated before sterile filtration, fill, and finish. In the case of split vaccine, the virus is split and the splitting agent is removed prior to formulation and sterile filtration at the expense of immunogenicity [3–6]. Split influenza vaccines are more commonly manufactured than whole inactivated virus (WIV) vaccines, because split vaccines have fewer side effects [7, 8]. Introduced almost 60 years ago, the initial splitting protocols were based on diethyl-ether extraction of the virus [9, 10]. However, diethyl-ether (ether) is volatile, posseses the risk of explosion, can cause irritation of the skin and eyes, and can lead to systemic organ damage after prolonged and repeated exposure. Manufacturers also had difficulty in determining HA concentration in the split vaccine using this method [11]. Therefore today, most split influenza vaccines are produced by using either deoxycholate (Afluria, Flulaval, Fluarix) or TritonX-100 (Fluzone).

Even though influenza vaccines have been used for ~50 years, several limitations still exist involving both their availability and their effectiveness [12]. Antigenic variation in circulating strains due to evolution of the viral HA and NA proteins results in immune evasion by preventing antibody binding and the subsequent neutralization of infection. Twice per year, the World Health Organiziation (WHO) [13] makes recommendations to influenza vaccine manufacturers and national governmental agencies as to which of the circulating strains should be included in the next hemisphere’s influenza season. Currently, two influenza A viral strains, representing the H1N1 and H3N2 subtypes are recommended, as well as two influenza B viral strains, representing the Yamagata and Victoria lineages (http://www.who.int/influenza/vaccines/virus/recommendations/en/). However, there is a need to develop influenza vaccine strategies that will elicit more broadly-reactive or more universal responses to recognize a larger number of circulating influenza variants within and across subtypes in any given season and over multiple seasons [1, 2].

To address the need for more broadly reactive influenza vaccines, our group has previously reported on the methodology of antigen design, termed computationally optimized broadly reactive antigen (COBRA), using multiple rounds of layered consensus building to generate influenza vaccine HA immunogens [14–21]. COBRA HA antigens are able to elicit potent, broadly reactive HA-specific antibody responses that protect against both vaccine selected and drift variant influenza strains. In this report, live viruses expressing COBRA HA antigens were used to generate inactivated split virus (IIV) vaccines. In contrast to previous studies in mice using IIV or virus-like particle (VLP) vaccines [22], the IIV vaccines used in this study elicited reduced antibody titers with limited breadth of hemagglutinination-inhibition (HAI) activity against panels of H1N1 or H3N2 influenza viruses in ferrets immunologically naïve to influenza. Nevertheless, these IIV vaccinated ferrets were protected from influenza challenge, and exhibited rapid reduction in virus shedding from the nasal mucosa following infection.

## Materials and methods

### Vaccine preparation and vaccinations

Fitch ferrets (*Mustela putorius furo*, female, 6 to 12 months of age), negative for antibodies to circulating influenza A (H1N1, H3N2) and influenza B viruses, were descented and purchased from Triple F Farms (Sayre, PA). Ferrets were pair housed in stainless steel cages (Shor-line, Kansas City, KS) containing Sani-Chips laboratory animal bedding (P.J. Murphy Forest Products, Montville, NJ). Ferrets were provided with Teklad Global Ferret Diet (Harlan Teklad, Madison, WI) and fresh water *ad libitum*. Ferrets (*n* = 8) were vaccinated with inactivated split IIV vaccines expressing either one of 2 H3N2 COBRA IIV vaccines (T7, T11), one of 4 wild-type IIV vaccines (HK/2014, Switz/2013, TX/2012, Uru/2007, Wisc/2005), one of the 3 H1N1 COBRA (X3, X6, or P1), or the wild-type CA/2009 IIV vaccine. The split virion vaccine was inactivated with 0.1% Beta-propiolactone (BPL) for 24 h between 18-22C and 1% Triton X-100 in the presence of 500mg/L Tween 80 that was stirred for 1 h at 20C [23]. The vaccines contained 15 μg of hemagglutinin antigen formulated with an emulsified squalene-in-water AF03 adjuvant (Sanofi Pasteur, Lyon, France) in a final 1:1 mixture with IIV (Table 1). Ferrets were boosted 28 days after initial vaccination. Blood was harvested from all anesthetized ferrets via the anterior vena cava prior to vaccination, and at days 28 and 56 post-initial vaccination. Serum was transferred to a centrifuge tube and centrifuged at 6000 rpm. Clarified serum was removed and frozen at −20 ± 5°C. Ferrets vaccinated with placebo consisted of phosphate-buffered saline (PBS), pH 7.4, formulated in a 1:1 mixture with AF03 [15, 16, 21]. All vaccines and placebo were stored in a refrigerator at a temperature between 2°C and 8°C until use.

**Table 1:** Vaccination schema of naïve ferrets.

		D0 and D28					
Group	N	Immunization	Subtype	Adjuvant	Dose	Volume	Route
1	8	PBS	N/A	AFO3	N/A	250uL antigen 250uL adjuvant	IM
2	8	A/Hong Kong/4801/2014 IIV	A(H3N2)	AFO3	15ug	250uL antigen 250uL adjuvant	IM
3	8	A/Switzerland/9715293/2013 IIV	A(H3N2)	AFO3	15ug	250uL antigen 250uL adjuvant	IM
4	8	A/Texas/50/2012 IIV	A(H3N2)	AFO3	15ug	250uL antigen 250uL adjuvant	IM
5	8	A/Uruguay/716/2007 IIV	A(H3N2)	AFO3	15ug	250uL antigen 250uL adjuvant	IM
6	8	A/Wisconsin/67/2005 IIV	A(H3N2)	AFO3	15ug	250uL antigen 250uL adjuvant	IM
7	8	COBRA T-7 IIV	A(H3N2)	AFO3	15ug	250uL antigen 250uL adjuvant	IM
8	8	COBRA T-11 IIV	A(H3N2)	AFO3	15ug	250uL antigen 250uL adjuvant	IM
							
9	8	A/California/07/2009 IIV	A(H1N1)	AFO3	15ug	250uL antigen 250uL adjuvant	IM
10	8	COBRA P-1 IIV	A(H1N1)	AFO3	15ug	250uL antigen 250uL adjuvant	IM
11	8	COBRA X-3 IIV	A(H1N1)	AFO3	15ug	250uL antigen 250uL adjuvant	IM
12	8	COBRA X-6 IIV	A(H1N1)	AFO3	15ug	250uL antigen 250uL adjuvant	IM

Twelve groups of naïve ferrets (n = 8) were vaccinated intra-muscularly (IM) with 500uL mixtures of IIV and AFO3 adjuvant at day 0 and at day 28. One group of ferrets received PBS vaccinations while others received either wild-type or COBRA H1N1 (P-1, X-6, X-3) or H3N2 (T-7, T-11) HA vaccines.

### Viruses and HA antigens

H1N1 and H3N2 viruses were obtained through either the Influenza Reagents Resource (IRR), BEI Resources, the Centers for Disease Control (CDC), or provided by Virapur, LLC (San Diego, CA, USA). Viruses were passaged once in the same growth conditions as they were received, in either embryonated chicken eggs or semi-confluent Madin-Darby Canine Kidney (MDCK) cell culture as per the instructions provided by the WHO [13]. Virus lots were titered with both guinea pig and turkey erythrocytes, and made into aliquots for single-use applications. The H3N2 vaccine panel includes the following egg passaged strains: A/Nanchang/933/1995 (Nan/95), A/Sydney/05/1997 (Syd/97), A/Panama/2007/1999 (Pan/99), A/Fujian/411/2002 (Fuj/02), A/New York/55/2004 (NY/04), A/Wisonsin/67/2005 (Wis/05), A/Brisbane/10/2007 (Bris/07), A/Perth/16/2009 (Per/09), A/Victoria/361/2011 (Vic/11), A/Texas/50/2012 (TX/12), A/Switzerland/9715293/2013 (Sz/13), A/Hong Kong/4801/2014 (HK/14), and A/Singapore/IFNIMH-16-0019/2016 (Sing/16).

A panel of 13 co-circulating MDCK cell passaged reassortant (7:1) H3N2 variants from the period of 2010–2016 included: A/Alabma/05/2010 (AL/10), A/Netherlands/009/2010 (NL/10), A/Hessen/5/2010 (Hes/10), A/Norway/1330/2010 (Nor/10), A/Madagascar/0648/2011 (Mad/11), A/Utah/12/2011 (Utah/11), A/Norway/1186/2011 (Nor/11), A/Athens/112/2012 (Ath/12), A/Jordan/30502/2012 (Jor/12), A/Minnesota/10/2012 (MN/12), A/Denmark/96/2013 (Den/13), A/Hong Kong/12/2014 (HK/12/14), A/Stockholm/28/2016 (Stock/16). Reassortant viruses were generated by using an eight-plasmid reverse genetics system as described previously [24, 25].

H1N1 egg passaged viruses used were A/Chile/1/1983 (Chile/83), A/Singapore/6/1986 (Sing/86), A/Texas/36/1991 (TX/91), A/Beijing/262/1995 (Bei/95), A/New Caledonia/20/1999 (NC/99) A/Solomon Islands/3/2006 (SI/06), A/Brisbane/59/2007 (Bris/07), A/California/07/2009 (CA/09), A/Michigan/45/2015 (Mich/15). The 9-member panel included viral antigens representing human viruses from 1983 to 2015. The P1, X6, and X3 HA rescued (7:1) viruses (PR8 core proteins and NA from CA/09) were detected by using a hemagglutination assay, and were fully sequenced to ensure the absence of unwanted mutations.

### Viral challenges of pre-immune vaccinated ferrets

On day 56 post-vaccination, ferrets were challenged intranasally with 5 × 10^4^ PFU of the CA/09 (H1N1) or 1 × 10^7^ PFU of the Wisc/05 (H3N2) virus in a volume of 1 ml. Ferrets were monitored daily for weight loss, disease signs, and death for 14 days after infection. Individual body weights and death were recorded for each group on each day post virus challenge. Experimental endpoints were defined as >20% weight loss. Nasal washes were performed by instilling 3 ml of PBS into the nares of anesthetized ferrets each day for 14 days after inoculation. Washes were collected and stored at −80°C until use. The University of Georgia Institutional Animal Care and Use Committee approved all experiments under the Animal Use Protocol #2016 02–011, which were conducted in accordance with the National Research Council’s *Guide for the Care and Use of Laboratory Animals*, The Animal Welfare Act, and the CDC/NIH’s *Biosafety in Microbiological and Biomedical Laboratories* guide.

### Hemagglutination-inhibition assay

The hemagglutination inhibition assay was used to assess functional antibodies, elicited by H3N2 vaccines, to the HA able to inhibit agglutination of guinea pig and as well as turkey erythrocytes. The protocols were adapted from the WHO laboratory influenza surveillance manual [13] and uses the host-species, guinea pig, that is frequently used to characterize contemporary H3N2 strains due to their preferential binding to alpha (2,6) linked sialic acid receptors [26, 27]. We also compared HAI results with turkey erythrocytes to compare whether there was a differential in HAI response depending on erythrocyte used. To inactivate nonspecific inhibitors, sera were treated with receptor-destroying enzyme (RDE) (Denka Seiken, Co., Japan) prior to being tested. Briefly, three parts of RDE was added to one part of sera and incubated overnight at 37°C. RDE was inactivated by incubation at 56°C for ∼30 min. RDE-treated sera were brought up to a final 1:10 mixture in physiological saline, and diluted in a series of twofold serial dilutions in 96-well v-bottom microtiter plates. An equal volume of each H3N2 virus, adjusted to approximately 8 hemagglutination units (HAU)/50 μl in the presence of 20nM Oseltamivir, was added to each well. The plates were covered and incubated at room temperature for 30 min, and then 0.75% guinea pig erythrocytes (Lampire Biologicals, Pipersville, PA, USA) in PBS were added. Red blood cells were stored at 4°C and used within 24 h of preparation. The plates were mixed by agitation and covered, and the RBCs were allowed to settle for 1 h at room temperature. The HAI titer was determined by the reciprocal dilution of the last well that contained non-agglutinated RBCs. Positive and negative serum controls were included for each plate. All ferrets were negative (HAI ≤ 1:10) for pre-existing antibodies to currently circulating human influenza viruses prior to vaccination, and seroprotection was defined as HAI titer >1:40 and seroconversion as a 4-fold increase in titer compared to baseline, as per the WHO and European Committee for Medicinal Products to evaluate influenza vaccines [28]; however, we often examined a more stringent threshold of >1:80. Ferrets were naïve and seronegative to influena at the time of vaccination, thus seroconversion and seroprotection rates are interchangeable in this study. For H1N1 vaccines, the procedure was modified such that serum and virus were incubated at room temperature for 20 min and then 0.8% turkey erythrocytes (Lampire Biologicals, Pipersville, PA) in PBS were added. The RBCs were stored at 4°C and used within 72 h of preparation. The remainder of the procedure was not altered.

### Plaque assay

MDCK and MDCK-SIAT1 cells were maintained in Dulbecco’s modified Eagle medium (DMEM) supplemented with penicillin-streptomycin, bovine serum albumin fraction V 7.5% solution, 25 mM HEPES buffer and 10% heat-inactivated fetal bovine serum (FBS, *Atlanta Biologicals*, GA, USA). All supplements except FBS were purchased from *Thermo Fisher Scientific*. For virus titering, a plaque assay was performed similarly to previously described protocols [16]. MDCK cells were seeded at (5 × 10^5^) in each well of a six-well plate. Samples were diluted (final dilution factors of 10^0^ to 10^−6^) and overlaid onto the cells in 100μl of DMEM supplemented with penicillin- streptomycin and incubated for 1 h with intermittent shaking every 15 minutes. Samples were removed, cells were washed twice, and medium replaced with 2 ml of L15 medium plus 0.8% agarose (Cambrex, East Rutherford, NJ, USA) and incubated for 72 h at 37°C with 5% CO2. Agarose was then removed and discarded. The cells were fixed with 10% buffered formalin, and then stained with 1% crystal violet for 15 min. The plates were then thoroughly washed in distilled water (dH_2_O) to remove excess crystal violet, before being air-dried. Next the number of plaques were counted, and the PFU per milliliter was calculated for each virus.

### Statistical analysis

Differences in weight loss, sickness score, and viral titers were analyzed by two-way ANOVA, followed by Bonferroni’s post-test for each vaccine group at multiple time points. Statistical significance was defined as *p*-value of 0.05. Statistical analyses were done using GraphPad Prism software.

## Results

### COBRA HA split inactivated virus vaccination of immunologically naïve ferrets

**Fig 1:**
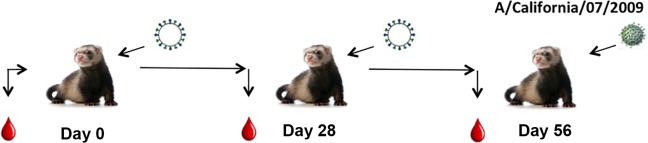
Schematic of IIV vaccination of naive ferrets. **Ferrets (n = 8) were vaccinated with IIV vaccine at day 0 and again at day 28. Blood was collected prior to initial infection and at days 28 and 56 post-vaccination**. At day 56, the ferrets were challenged with either A/California/07/2009 (H1N1) virus (5x10^4^ PFU) or A/Wisconsin/67/2005 (H3N2) virus (1x10^7^ PFU) and monitored for morbidity and mortality for 14 days post-infection. Nasal washes were collected at days 0, 1, 3, 5, and 7 to assess viral titers.

**Fig 2:**
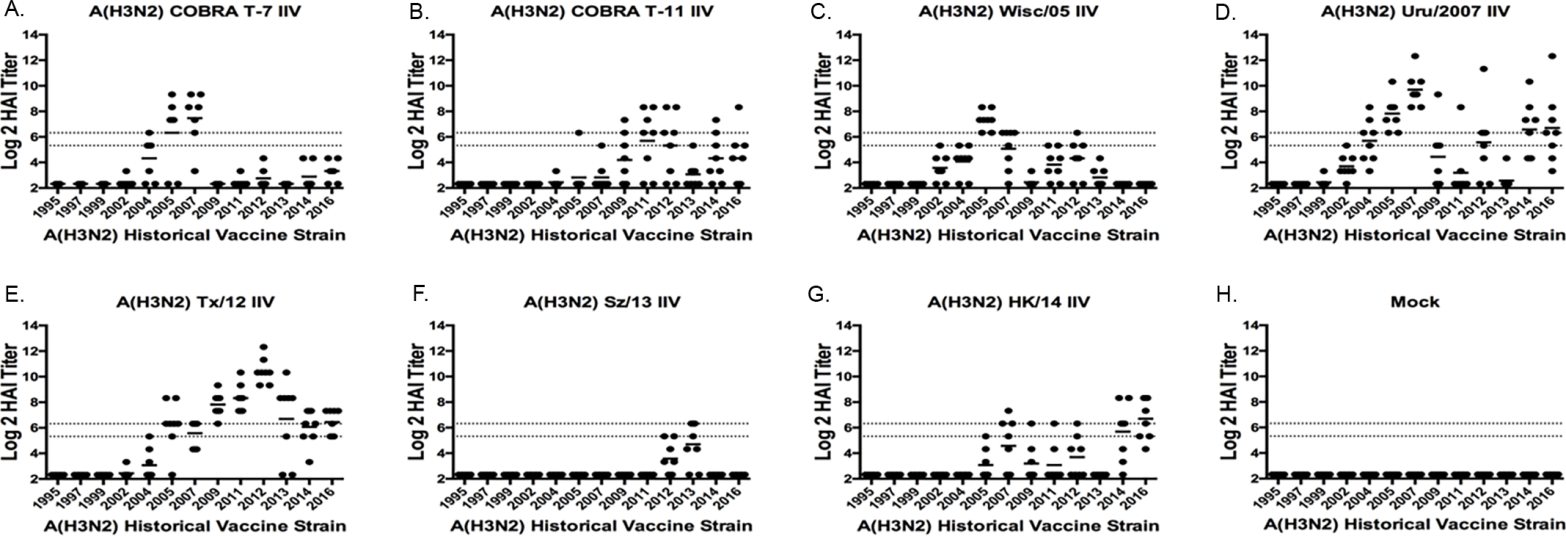
Hemagglutination inhibition serum antibody titers induced by vaccination of ferrets with H3N2 IIV vaccines expressing COBRA HA antigens or HA proteins from wild-type viruses. HAI titers were determined for each group of immunologically naïve ferrets (n = 8) vaccinated two times (days 0 and 28) with 1 of the 2 COBRA H3N2 IIV vaccines (T7 or T11) or H3N2 VLP vaccines expressing wild-type HA proteins from Wisc/05, Uru/07, TX/12, Sz/13, HK/14 against a panel of 13 H3N2 influenza viruses. Values are the individual animal HAI titers from antisera collected on day 56. The two dotted lines indicates the 1:40–1:80 HAI titer range. (A) T6 IIV; (B) T11 IIV; (C) Wisc/05 IIV; (D) Uru/07 IIV; (E) TX/12 IIV; (F) Sz/13 IIV; (G) HK/14 IIV; (H) Mock.

Ferrets (n = 8/group) were vaccinated with split inactivated virus (IIV) vaccines containing COBRA or wild-type HA proteins representing the H3 subtype. Previous studies have described the COBRA methodology using 6,340 human HA amino acid sequences to design H3 HA sequences to account for the evolution of H3N2 influenza viruses isolated in humans over the past 45 years [16, 21]. Two of these candidates, identified as T7 IIV and T11 IIV, were used as vaccine antigens in naïve ferrets, and were compared to ferrets vaccinated with IIV vaccines containing wild-type HA antigens isolated from five contemporary vaccine strains (Wisc/05, Uru/07, TX/12, Sz/13, HK/14) (Fig 1). As previously described [21], the T7 COBRA HA antigen was designed using input HA sequences from H3N2 viruses isolated between 2002–2010 and the T11 COBRA HA sequence was designed with input HA sequenes from H3N2 viruses isolated between 2011–2013. Antisera collected from ferrets vaccinated with T7 IIV had HAI activity against 3 of the 12 H3N2 viruses in the panel (Fig 2A). T7 IIV elicited antibodies with an HAI titer of 1:40 in 50% or more of the ferrets against NY/04, Wisc/05, and Uru/07 (Table 2). Ferrets vaccinated with T11 IIV had antisera with HAI activity against four (Perth/09, Vic/11, TX/12, and HK/14) of the 12 viruses in the panel (Fig 2B and Table 2). Ferrets vaccinated with one of the five wild-type HA IIV vaccines generally had the highest HAI activity against its homologous virus, *i*.*e*. Wisc/05 elicited antibodies with the highest HAI titers against Wisc/05. The Wisc/05 IIV vaccine elicited antibodies with an average HAI titer above 1:40 against only 1 virus, the Wisc/05 virus (Fig 2C). The Uru/07 IIV vaccine elicited antibodies with an average HAI titer of 1:40 against 5 strains in the panel (Fig 2D), with all 8 ferrets having HAI activity against Wisc/05 and Uru/07 (Table 2). TX/12 IIV vaccinated ferrets had HAI activity against 7 of the 12 strains (Fig 2E). Ferrets vaccinated with Sz/13 had no HAI activity against any of the viruses at a 1:40 titer, but recognized the homologous strain Sz/13 at a higher average titer than any other strain in the panel. Ferrets vaccinated with HK/14 had antibodies against only one virus, the homologous HK/14, at a 1:40 titer (Fig 2F and 2G).

**Table 2:** Historical vaccine strain panel (1995–2016) HAI heat map.

**1:40 Cutoff**	Nan/95	Syd/97	Pan/99	Fuj/02	NY/04	Wisc/05	Bris/07	Perth/09	Vic/11	Tx/12	Switz/13	HK/14	Sing/16
**PBS**	0	0	0	0	0	0	0	0	0	0	0	0	0
**HK/14**	0	0	0	0	0	1	4	1	1	2	0	5	7
**Switz/13**	0	0	0	0	0	0	0	0	0	2	4	0	0
**Tx/12**	0	0	0	0	1	7	5	8	8	8	6	7	8
**Uru/07**	0	0	0	1	4	8	8	4	1	5	0	5	0
**Wisc/05**	0	0	0	1	2	8	5	0	2	3	0	0	0
**COBRA T7**	0	0	0	0	4	5	6	0	0	0	0	0	6
**COBRA T11**	0	0	0	0	0	1	1	3	5	5	1	3	3
**1:80 Cutoff**	Nan/95	Syd/97	Pan/99	Fuj/02	NY/04	Wisc/05	Bris/07	Perth/09	Vic/11	Tx/12	Switz/13	HK/14	Sing/16
**PBS**	0	0	0	0	0	0	0	0	0	0	0	0	0
**HK/14**	0	0	0	0	0	0	3	1	1	1	0	5	5
**Switz/13**	0	0	0	0	0	0	0	0	0	0	3	0	0
**Tx/12**	0	0	0	0	0	6	5	8	8	8	5	5	5
**Uru/07**	0	0	0	0	3	8	8	1	1	4	0	5	0
**Wisc/05**	0	0	0	0	0	8	4	0	0	1	0	0	0
**COBRA T7**	0	0	0	0	1	5	6	0	0	0	0	0	5
**COBRA T11**	0	0	0	0	0	1	0	2	5	4	0	2	1

Numbers represent the number of animals from each group that achieved an antibody titer ≥1:40 in the (top table) and ≥1:80 (bottom table) against 13 histoical H3N2 vaccine strains. Colors range from red representing 0 animals achieving the designated titer to dark green representing when all 8 animals surpassed the antibody titer cutoff.

### HAI activity against co-circulating drift variant viruses

In order to demonstrate the breadth of antibody activity against co-circularing H3N2 influezna strains between 2010 and 2016, HAI activity was assessed from the collected antisera following vaccination. Human H3 hemagglutinin sequences were identified to represent different H3N2 influenza phylogenetic clusters and thirteen representative drift viruses were used in a panel to assess the breadth of HAI activity elicited by the IIV vaccines as previously described [21]. Ferrets vaccinated with IIV vaccines expressing the HA protein from HK/14, Sz/13, or Wisc/05 had HAI activity against 3–4 viruses out of the 13 H3N2 viruses in the panel, which was similar to the HAI activity in ferrets vaccinated with COBRA T7 (Table 3). Ferrets vaccinated with the Uru/07 IIV vaccines had antibodies with HAI activity against 7 of the 13 strains, and antisera from ferrets vaccinated with TX/12 IIV had HAI activity against all 13 strains (Table 3). Ferrets vaccinated with the T11 COBRA HA IIV vaccine had antisera with HAI activity against all but one strain in the panel, Stock/16. The HAI titers elicited by TX/12 IIV in naïve ferrets were 4–8 fold higher than HAI titers elicited by T11 COBRA IIV vaccine. However, T11 COBRA IIV elicited HAI titers that were 4–32 fold higher against the panel of H3N2 drift variant viruses than any of the other wild-type HA IIV vaccines (Table 3).

**Table 3:** Log(2) GMT heatmap: H3 co-circulating strains panel (2010–2016).

	Log(2) GMT												
	Norway/10	Alabama/10	Hessen/10	Netherlands/10	Norway/11	Madagascar/11	Utah/11	Athens/12	Jordan/12	Minnesota/12	Denmark/13	Hong Kong/12/14	Stockholm/16
PBS	2.32	2.32	2.32	2.32	2.32	2.32	2.32	2.32	2.32	2.32	2.32	2.32	2.32
HK/14	4.57	4.45	4.32	4.82	3.70	6.20	4.70	5.95	4.57	7.57	4.95	5.45	4.32
Sz/13	3.70	3.70	3.32	3.95	3.07	5.82	4.20	4.57	3.32	6.82	4.82	5.32	3.57
Tx/12	8.57	8.32	9.20	8.57	8.82	9.95	8.07	9.57	9.33	9.45	9.20	8.95	6.70
Uru/07	5.95	5.82	5.07	6.33	4.70	8.07	5.20	5.45	7.07	6.82	4.82	5.45	3.57
Ws/05	4.07	3.57	4.07	4.20	3.95	5.95	4.70	5.32	4.70	7.45	4.32	4.95	3.82
COBRA T7	2.75	3.61	3.04	4.46	2.46	5.75	3.18	3.46	4.75	8.75	2.75	4.32	3.18
COBRA T11	6.82	6.57	6.57	6.82	7.32	7.95	6.57	8.07	7.57	7.95	6.57	6.82	3.95

Colored cells represent groups that achieved a GMT above 5.32 which correlate to an average antibody titer ≥1:40. Values closest to 5.32 are colored yellow and become more green as the values increase. Cells with no color represent groups that did not achieve a GMT ≥5.32.

### Vaccination of ferrets with IIV vaccines expressing H1 HA antigens

**Fig 3:**
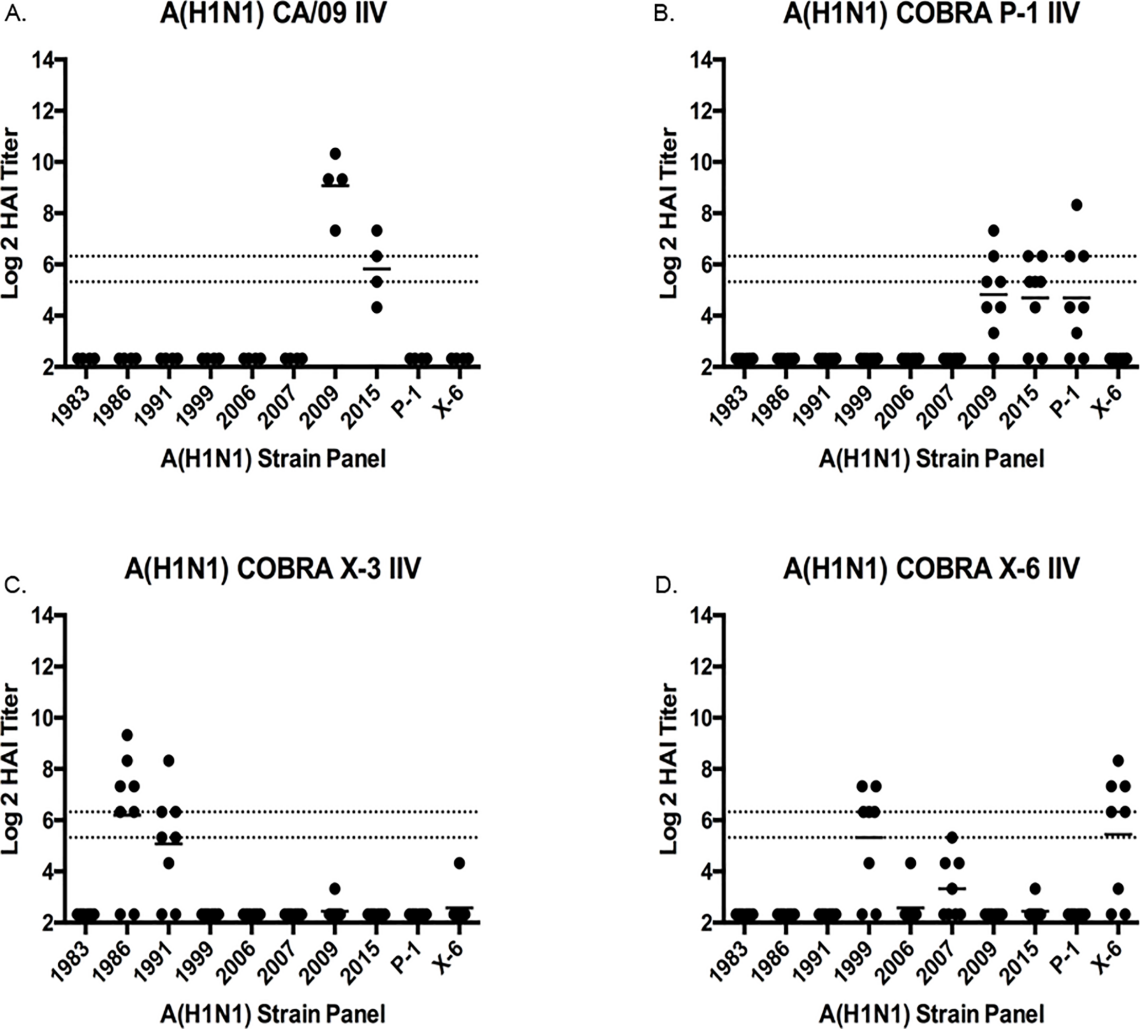
Hemagglutination inhibition serum antibody titers induced by vaccination of ferrets with H1N1 IIV vaccine expressing COBRA HA antigens or HA proteins from wild-type viruses. HAI titers were determined for each group of immunologically naïve ferrets (n = 8) vaccinated two times (days 0 and 28) with 1 of the 3 COBRA H1N1 IIV vaccines (P1, X3 or X6) or H1N1 IIV vaccines expressing wild-type HA proteins from CA/09 against a panel of 8 H1N1 influenza viruses. Values are the individual animal HAI titers from antisera collected on day 56. The two dotted lines indicates the 1:40–1:80 HAI titer range. (A) CA/09 IIV; (B) P1 IIV; (C) X3 IIV; (D) X6 IIV.

To extend these observations, ferrets were vaccinated with IIV vaccines expressing H1 HA antigens, one IIV expressed as the HA from the wild-type (CA/09) influenza virus and three IIV vaccines expressed as H1 COBRA HA antigens (P1, X3, or X6) (Fig 3). These three candidate COBRA HA vaccines were designed based on wild-type sequences of H1N1 viruses spanning the past 100 years, including modern pandemic H1N1 isolates. Previous studies (Carter et al., 2016, JVI) demonstrated that these three H1 COBRA HA proteins had the broadest hemagglutination inhibition activity against a panel of 17 H1N1 viruses [22]. Ferrets (n = 8/group) vaccinated with CA/09 IIV elicited high titer antibodies with HAI activity against (average titer is 1:320) the homologous CA/09 virus (Fig 3A). About half of the ferrets vaccinated with the P1 COBRA IIV vaccine seroconverted and had antisera with HAI activity against its homologous antigens, but did not elicit HAI activity against any of the other H1N1 viruses in the panel (Fig 3B). Ferrets vaccinated with the X3 IIV vaccine elicited HAI activity against Sing/86 and TX/91 (Fig 3C), and ferrets vaccinated with X6 IIV had HAI activity against the homologous X6 virus as well as the NC/99 virus with low HAI titers against Bris/07 (Fig 3D). Similar results were observed in naïve ferrets vaccinated with virus-like particles (VLP) expressing the same wild-type and COBRA HA antigens (15).

### Vaccinated ferrets challenged with influenza viruses

**Fig 4:**
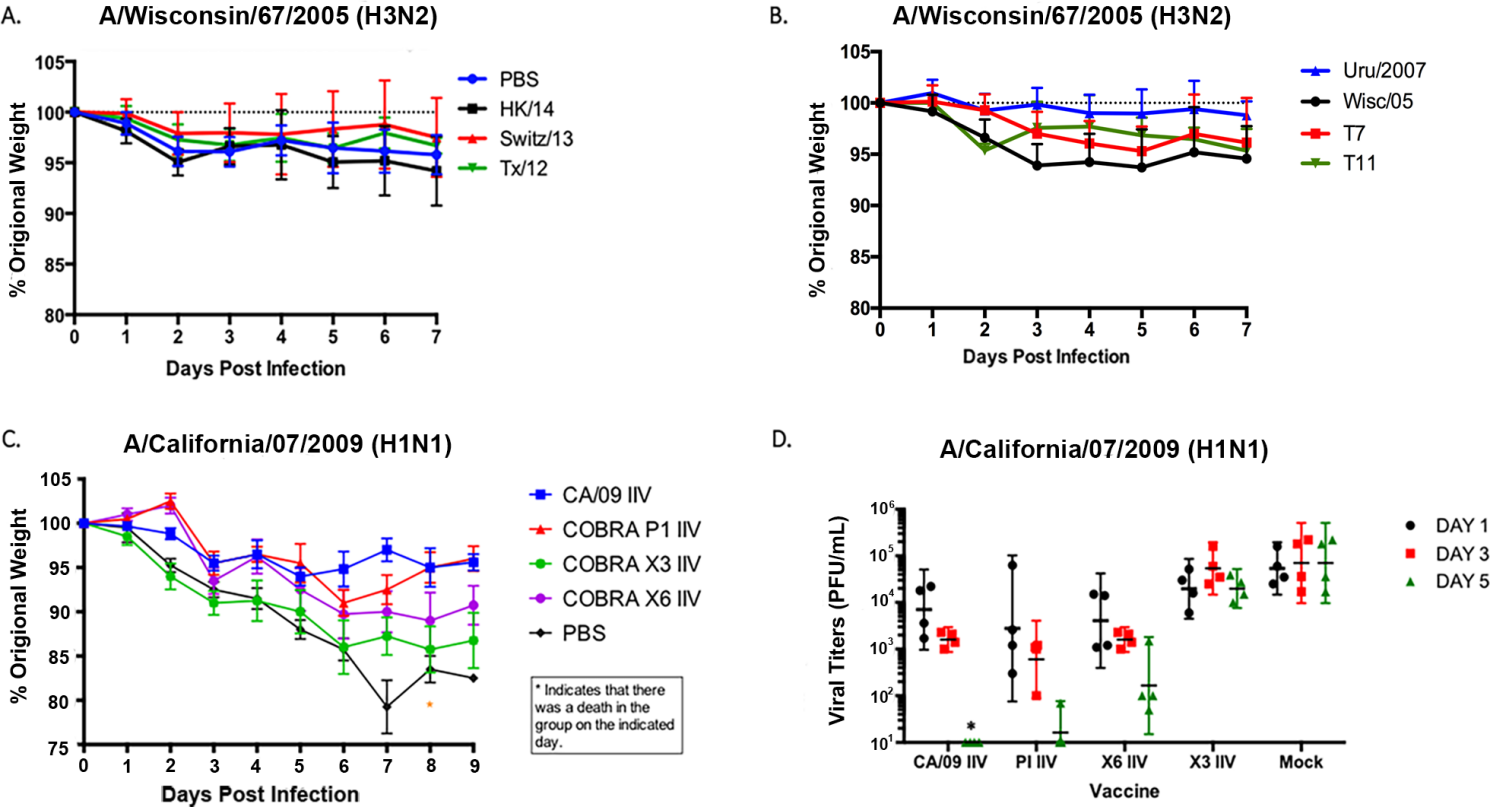
Challenge of vaccinated ferrets with influenza viruses. Vaccinated or unvaccinated ferrets were infected with A/Wisconsin/67/2005 (A and B) or A/California/07/2009 (C) influenza viruses. (A-C) Ferrets were monitored daily for weight loss over an 8-day observation period. Values are average percentages of original weight plus the SEM (error bars). (D) Viral titers were determined from nasal washes infected with A/California/07/2009 and collected at days 1, 3, and 5 post-challenge. Each ferret is represented with a symbol. Lines indicate mean virus titers with the standard deviations (SD). The values for CA/09, P1, and X6 are all significant (p>0.01) compared to the values at each timepoint for X3 and Mock. CA/09 was significant compared to P1 and X6 at day 3 post-infection (p>0.05). Significance was determined using paired or unpaired t tests (*p>0.05; **p >0.*01*).

To determine whether these IIV vaccines protected against viral challenge, regardless of the HAI titers in the elicited antisera, ferrets were challenged with either H1 or H3 influenza viruses (Fig 4). Ferrets vaccinated with H3 IIV vaccines were challenged with Wisc/05 (1×10^7^ PFU/ml) at week 8 post-vaccination. All ferrets lost less than 5% weight regardless if they were vaccinated or not (Fig 4A and 4B). Low titers of virus (1 x 10^3^ PFU/ml) were recovered from the nasal washes from the mock vaccinated ferrets infected with the H3N2 virus, and no virus was detectable in nasal washes from vaccinated ferrets. Ferrets vaccinated with H1 IIV vaccines were challenged with CA/09 virus (5×10^4^ PFU/ml). Non-vaccinated ferrets (mock) had a rapid drop in weight, losing 15–20% of their body weight by day 7 post-infection (Fig 4C). These ferrets showed signs of morbidity, including lethargy, sneezing, and nasal discharge, as previously described for novel H1N1 infection [29]. In contrast, ferrets vaccinated with the homologous CA/09 IIV or the P1 IIV vaccines lost less than 5–6% of their original body weight over the 10 days of observation. Ferrets vaccinated with the X6 IIV lost ~10% of their original body weight by day 6 post-infection, and ferrets vaccinated with X3 IIV lost ~15%. All X6 and X3 IIV vaccinated ferrets maintained that weight for 2–3 days and then began to recover (Fig 4C).

Naïve animals had high nasal wash viral titers on days 1–5 post-infection (~2×10^5^ pfu/ml) (Fig 4D). In addition, ferrets vaccinated with X3 IIV also had a sustained viral nasal wash titer between days 1–5 post-infection. In contrast, ferrets vaccinated with either CA/09 IIV, P1 COBRA IIV, or X6 COBRA IIV had a peak nasal wash viral titer at day 1 (~1×10^4^ pfu/ml) that rapidly declined to low or undetectable levels by day 5 post-infection (Fig 4D).

## Discussion

In this study, reassortant viruses expressing H1 and H3 COBRA HA antigens were generated by combining the desired HA segment with the 7 segments from the A/Puerto Rico/8/1934 virus. These viruses were subsequently amplified and used to generate inactivated split influenza vaccines (IIV). The first goal was to evaluate these IIV vaccines in naïve ferrets for elicitation of antibodies. Previously, these COBRA HA antigens, delivered to the immune system of mice on the surface of a virus-like particle vaccines, which elicited high titer antibodies against a panel of historical influenza A strains [16, 21]. We hypothesized that delivering these same antigens as an IIV vaccine would elicit similar antibody responses in ferrets, and protect these animals from influenza virus challenge.

**Fig 5:**
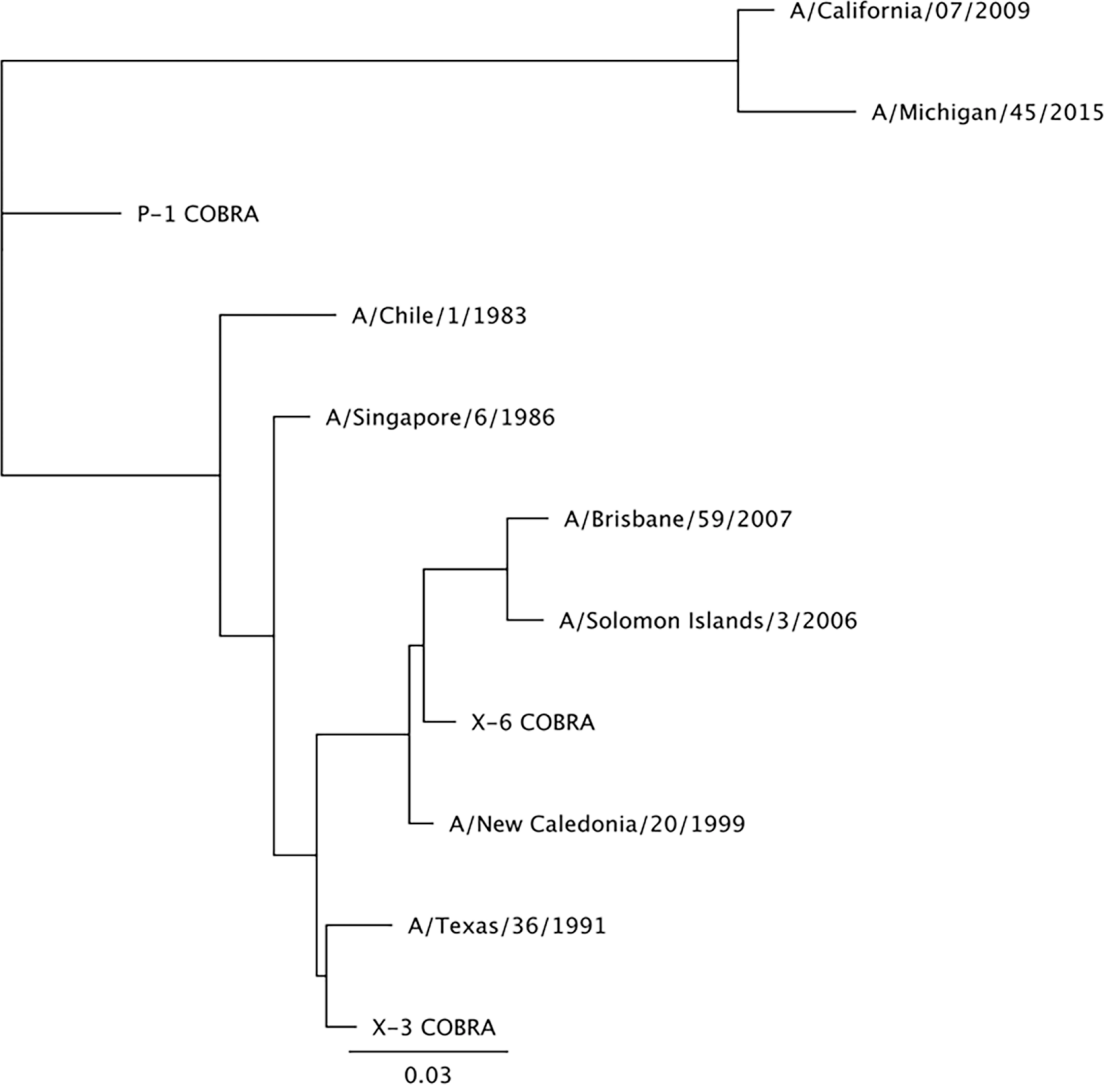
Phylogenetic tree of H1 HA sequences. The unrooted phylogenetic tree was inferred from HA amino acid sequences derived from 11 H1 HA isolates and COBRA HA using the maximum likelihood method. Sequences were aligned with MUSCLE 3.7 software, and the alignment was refined by Gblocks 0.91b software. Phylogeny was determined using the maximum likelihood method with PhyML software. Trees were rendered using TreeDyn 198.3 software [32].

**Fig 6:**
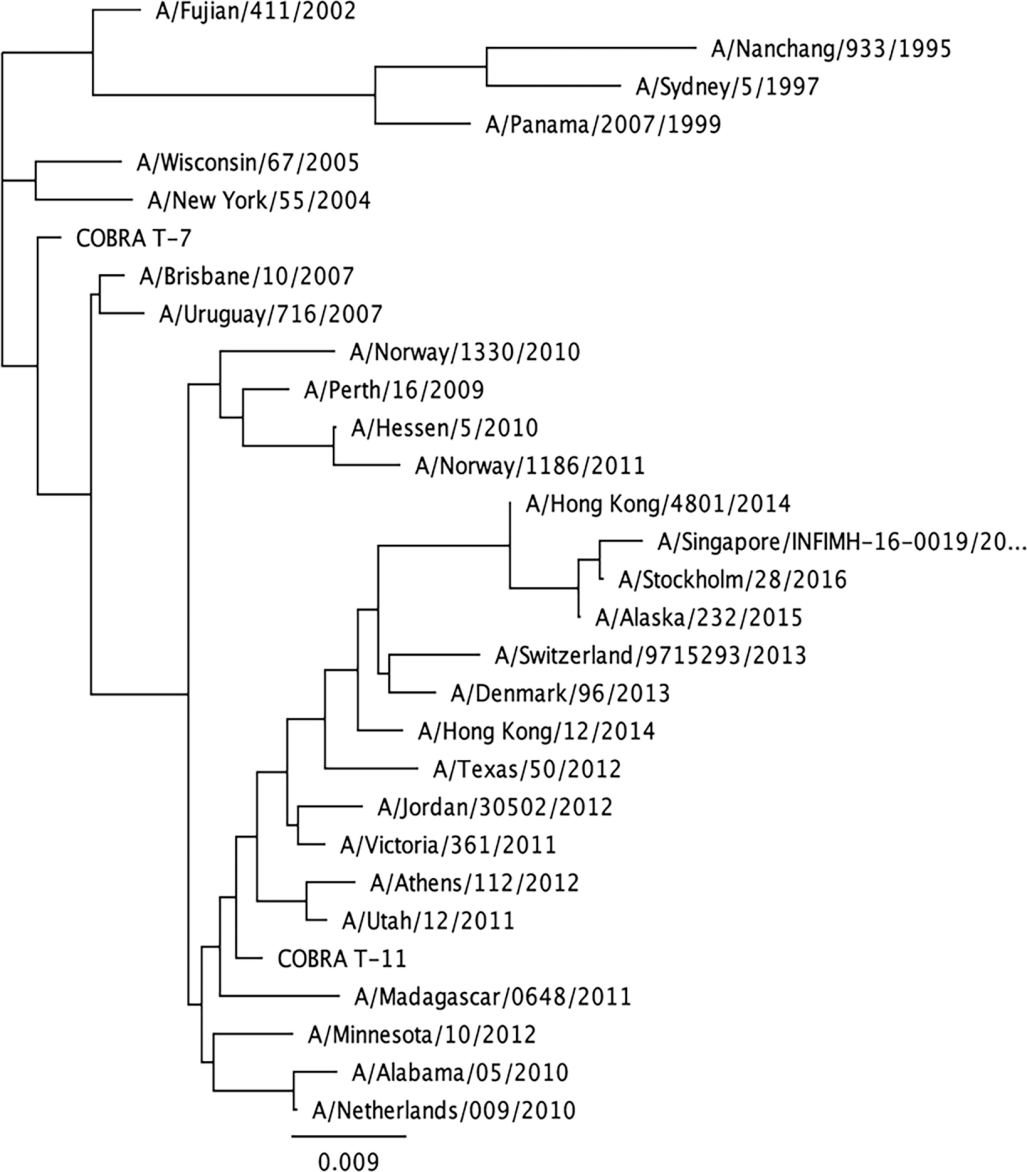
Phylogenetic tree of H3 HA sequences. The unrooted phylogenetic tree was inferred from HA amino acid sequences derived from 30 H3 HA and COBRA HA sequences using the maximum likelihood method. Sequences were aligned with MUSCLE 3.7 software, and the alignment was refined by Gblocks 0.91b software. Phylogeny was determined using the maximum likelihood method with PhyML software. Trees were rendered using TreeDyn 198.3 software [32].

The two H3 and three H1 COBRA HA antigens were designed to cover multiple time periods and antigenic spaces based on their date of isolation, as previously described [16, 21]. Their phylogenetic relationship to wild-type HA sequences are associated with the era of their input sequences, and are similar to the vaccine strains from that epoch (Figs 5 and 6). The two H3 COBRA HA vaccines represented various input sequences from viruses isolated between 2002 to 2013. The three H1 COBRA antigens including the X3 COBRA HA were designed using sequences from seasonal H1 viruses isolated over a 30-year period (1978 to 2008), the X6 COBRA HA was based upon sequences representing the 10 years prior to the 2009 pandemic and the 4 years after the pandemic (2009 to 2012). The P1 COBRA HA sequence covers the H1 antigenic space of viruses isolated from 1933 to 1957 and 2009 to 2011, as well as swine sequences isolated from 1931 to 1998 [16].

The T7 COBRA IIV vaccine was more effective at eliciting antibodies with HAI activity against H3N2 viruses from the mid-2000s, and the T11 COBRA IIV vaccine was more effective at eliciting antibodies with HAI activity against H3N2 viruses from the late-2000s through 2016. These results were similar to those obtained from mice vaccinated with VLPs expressing these same H3 COBRA HA antigens [21]. Ferrets vaccinated with IIV vaccines expressing either the Uru/07 or TX/12 HA antigens were the most effective at eliciting antibodies with HAI activity against the largest number of H3N2 viruses in the panel (6–8 viruses) compared to IIV vaccines with Sz/13 or HK/14 HA proteins. As previously reported [21], the Uru/07, and particularly the TX/12 HA antigens are more closely related to the various co-circulating drift variant viruses than the other H3N2 viruses. Similar results were observed in ferrets using the H1 IIV vaccines, which elicited antibodies that had a similar pattern of recognition when compared to antibodies elicited by H1 COBRA VLP vaccines in mice [16]. Both H1 and H3 IIV vaccines elicited the highest titers against the homologous virus. Overall, these vaccines were effective at eliciting antibodies with HAI breadth; however, unlike mice, not all the ferrets seroconverted to non-homologous viruses in the panel. Thus, the average HAI titer against a specific virus was lower overall in this study compared to the mice vaccinated with COBRA HA VLP vaccines [16, 21].

In order to address if the lower titers, and albeit reduced recognition of viruses in the panel by the vaccine elicited antibodies, was a result of expressing the COBRA HA antigens in an IIV formulation, naïve ferrets were also vaccinated with VLP vaccines. VLP vaccinated ferrets had statistically similar HAI titers against the same panel of H1 and H3 viruses as antibodies elicited in ferrets vaccinated with IIV versions of these same vaccines. In contrast to BALB/c mice, ferrets are outbred animals, and this may have contributed to the inconsistent rates of seroconversion and spread in the HAI titers within a group of ferrets vaccinated with the same vaccine. Recently, our group demonstrated that priming naive ferrets with historical seasonal influenza viruses, and then vaccinating these primed ferrets with broadly reactive H1 or H3 COBRA HA based VLP vaccines boosted pre-existing antibodies induced by the wild-type influenza virus infections. These COBRA HA antigens induced antibodies with HAI activity against multiple antigenically distinct H1N1 and H3N2 viral variants [15, 30]. Therefore, the IIV versions of these vaccines may perform equally as well in immunologically primed ferrets as VLP vaccines. Most pre-clinical studies use immunologically naïve animals to test new influenza vaccines and adjuvant formulations. However, most humans are not immunologically naïve to influenza virus and have pre-existing antibodies elicited by past vaccinations and infections (14). Recently, the National Institute of Allergy and Infectious Diseases (NIAID) announced as one of its highest priorities the development of a universal influenza vaccine that would provide long-lasting protection against multiple strains of influenza virus [31]. The three main areas for the development of these broadly reactive/universal influenza vaccines are focused on 1) transmission, natural history, and pathogenesis studies using prospective cohorts, 2) influenza immunity and correlates of immune protection, and 3) strategies for rational vaccine design to elicit broad, protective immune responses. We propose that using only immunologically naïve animals, particularly ferrets, for assessment of new vaccine candidates, adjuvants, and regimens, may not accurately assess the effectiveness of these vaccines. Both scientists and vaccine developers should use both naïve and pre-immune animal models to assess the overall effectiveness of these novel vaccine candidates. The results of this study indicate that to meet the NIAID strategic plan for development of these next generation of influenza vaccines, multiple *in vitro* and *in vivo* models will need to be used to assess vaccine formulations, and downselect the candidates to those with the most likely chance of success in human clinical trials.
