# Correction: Split inactivated COBRA vaccine elicits protective antibodies against H1N1 and H3N2 influenza viruses

**DOI:** 10.1371/journal.pone.0210043

**Published:** 2018-12-27

**Authors:** James D. Allen, Satyajit Ray, Ted M. Ross

There is an error in Table 2. The coloring of cells in the table is incorrect. The coloring should appear as below. Additionally, “historical” is misspelled in the published table’s footnote.

**Table 2:** Historical vaccine strain panel (1995–2016) HAI heat map.

**1:40 Cutoff**	**Nan/95**	**Syd/97**	**Pan/99**	**Fuj/02**	**NY/04**	**Wisc/05**	**Bris/07**	**Perth/09**	**Vic/11**	**Tx/12**	**Switz/13**	**HK/14**	**Sing/16**
**PBS**	0	0	0	0	0	0	0	0	0	0	0	0	0
**HK/14**	0	0	0	0	0	1	4	1	1	2	0	5	**7**
**Switz/13**	0	0	0	0	0	0	0	0	0	2	4	0	0
**Tx/12**	0	0	0	0	1	7	5	8	8	8	6	7	8
**Uru/07**	0	0	0	1	4	8	8	4	1	5	0	5	0
**Wisc/05**	0	0	0	1	2	8	5	0	2	3	0	0	0
**COBRA T7**	0	0	0	0	4	5	6	0	0	0	0	0	6
**COBRA T11**	0	0	0	0	0	1	1	3	5	5	1	3	3
**1:80 Cutoff**	**Nan/95**	**Syd/97**	**Pan/99**	**Fuj/02**	**NY/04**	**Wisc/05**	**Bris/07**	**Perth/09**	**Vic/11**	**Tx/12**	**Switz/13**	**HK/14**	**Sing/16**
**PBS**	0	0	0	0	0	0	0	0	0	0	0	0	0
**HK/14**	0	0	0	0	0	0	3	1	1	1	0	5	5
**Switz/13**	0	0	0	0	0	0	0	0	0	0	3	0	0
**Tx/12**	0	0	0	0	0	6	5	8	8	8	5	5	**5**
**Uru/07**	0	0	0	0	3	8	8	1	1	4	0	5	0
**Wisc/05**	0	0	0	0	0	8	4	0	0	1	0	0	0
**COBRA T7**	0	0	0	0	1	5	6	0	0	0	0	0	5
**COBRA T11**	0	0	0	0	0	1	0	2	5	4	0	2	1

Numbers represent the number of animals from each group that achieved an antibody titer ≥1:40 in the (top table) and ≥1:80 (bottom table) against 13 historical H3N2 vaccine strains. Colors range from red representing 0 animals achieving the designated titer to dark green representing when all 8 animals surpassed the antibody titer cutoff.
