# Expression of Concern: Split inactivated COBRA vaccine elicits protective antibodies against H1N1 and H3N2 influenza viruses

**DOI:** 10.1371/journal.pone.0297253

**Published:** 2024-01-10

**Authors:** The PLOS ONE Editors

The article’s Competing Interests statement is updated to: “As is noted in the article’s Funding statement, this work, including the efforts of TMR, was funded by Sanofi Pasteur (CROA UGA 001). SR is affiliated with Sanofi-Pasteur, Inc. and produced the IIV vaccines expressing COBRA HA proteins. The corresponding author (TMR) stated that the vaccines used in the study were not licensed by Sanofi Pasteur and that SR was not involved in the design of the study, performing the animal studies, assays, or analysis of the results.”

Contrary to the declaration in the Data Availability statement, the original raw data files supporting the article’s results were not provided with the article. A committee at the University of Georgia stated that they could not locate verifiable primary data sources or other records underlying the article [1, 2].

Since the original raw data files supporting the article’s results are no longer available, this article [1, 2] does not comply with the PLOS Data Availability Policy. The *PLOS ONE* Editors, therefore, issue this Expression of Concern.
